# A Privacy-Preserving Audit and Feedback System for the Antibiotic Prescribing of General Practitioners: Survey Study

**DOI:** 10.2196/31650

**Published:** 2022-07-13

**Authors:** Kassaye Yitbarek Yigzaw, Taridzo Chomutare, Rolf Wynn, Gro Karine Rosvold Berntsen, Johan Gustav Bellika

**Affiliations:** 1 Norwegian Centre for E-health Research University Hospital of North Norway Tromsø Norway; 2 Institute of Clinical Medicine UiT The Arctic University of Norway Tromsø Norway; 3 Divison of Substance Use and Mental Health University Hospital of North Norway Tromsø Norway; 4 Department of Community Medicine UiT The Arctic University of Norway Tromsø Norway

**Keywords:** learning health care system, feedback, antimicrobial stewardship, quality improvement, privacy, electronic health record, antibiotics, prescription, patient privacy, clinical setting

## Abstract

**Background:**

Antibiotic resistance is a worldwide public health problem that is accelerated by the misuse and overuse of antibiotics. Studies have shown that audits and feedback enable clinicians to compare their personal clinical performance with that of their peers and are effective in reducing the inappropriate prescribing of antibiotics. However, privacy concerns make audits and feedback hard to implement in clinical settings. To solve this problem, we developed a privacy-preserving audit and feedback (A&F) system.

**Objective:**

This study aims to evaluate a privacy-preserving A&F system in clinical settings.

**Methods:**

A privacy-preserving A&F system was deployed at three primary care practices in Norway to generate feedback for 20 general practitioners (GPs) on their prescribing of antibiotics for selected respiratory tract infections. The GPs were asked to participate in a survey shortly after using the system.

**Results:**

A total of 14 GPs responded to the questionnaire, representing a 70% (14/20) response rate. The participants were generally satisfied with the usefulness of the feedback and the comparisons with peers, as well as the protection of privacy. The majority of the GPs (9/14, 64%) valued the protection of their own privacy as well as that of their patients.

**Conclusions:**

The system overcomes important privacy and scaling challenges that are commonly associated with the secondary use of electronic health record data and has the potential to improve antibiotic prescribing behavior; however, further study is required to assess its actual effect.

## Introduction

Antibiotic stewardship is an issue that has recently come under scrutiny in Norway [1] and internationally [2], especially in general practices, which is where most antibiotic prescribing occurs [3]. The inappropriate use of antibiotics is thought to be an important factor in the development of antimicrobial resistance. Although a number of guidelines define when antibiotic use is warranted, defining *appropriate use* is difficult, unless there is clear agreement on the etiology of an infection [4]. There are diverse and complex reasons for the overuse of antibiotics, and consequently, several strategies have been designed to combat this problem [5]. There have been increasing calls by government agencies, such as the US Centers for Disease Control and Prevention, to embrace regular tracking and reporting (audits and feedback) as two of the core elements of stewardship programs [6].

Audits and feedback [7] can incorporate behavioral science elements, that is, clinical performance can be reviewed and compared among peers, and this has been widely reported as effective in reducing the inappropriate prescribing of antibiotics [1,2,8,9]. Despite strong support in the literature, there are at least two important factors that make audits and feedback hard to implement in real-life settings or make them ineffective altogether. First, without proper privacy safeguards, the risk of breaching regulatory compliance with regard to health data confidentiality increases. Additionally, underperforming general practitioners (GPs) may feel exposed and view the exercise as punitive [10]. The second factor, scalability, is important for efficiently analyzing vast amounts of distributed electronic health record (EHR) data to facilitate the regular scheduling of audit and feedback (A&F) programs. Regular scheduling is an important factor, since evidence shows that if audits and feedback are stopped, their benefits are likely to dissipate [11,12].

This study evaluates a scalable, privacy-preserving A&F system [13] in clinical settings. The system was deployed in three GP offices and used to generate feedback for 20 GPs on their antibiotic prescribing for selected respiratory tract infections (RTIs), which was viewed in comparison to the average performance of peers. The objective is to evaluate the GPs’ sentiments toward (1) the privacy protections that the system offers, (2) the accuracy and appropriateness of using personal feedback as the basis for comparisons with peers, and (3) self-efficacy and the potential of the intervention to improve the prescribing of antibiotics.

## Methods

### Privacy-Preserving System for Audits and Feedback

The privacy-preserving system contains software components that are deployed at each health institution. The system extracts data daily from the local EHR system and transforms and loads the data into a database that conforms to a common data model [14].

A third party (denoted as a *coordinator*) aids the system without learning private information and is trusted to follow protocol specifications. This is a standard security model known as the *honest-but-curious adversarial model* [15]. In this study, the Norwegian Centre for E-health Research acted as the coordinator. The coordinator accepts queries for the aggregated performance indicators of GPs across health care institutions. The system uses privacy-preserving distributed data mining techniques [16-18] for executing statistical queries on the combined data of health care institutions without allowing any party to view the private data that health care institutions compute locally. Statistical results on the combined data of multiple health care institutions are not considered sensitive information and are therefore stored at the coordinator and retrieved through a web service.

The feedback report is generated locally by combining local personal indicators and the aggregated indicators retrieved from the coordinator. Access to the feedback report is restricted to the respective GP and is provided through a web client or email as an encrypted PDF file, and the decryption key is sent to a mobile phone. We refer interested readers to a study by Yigzaw et al [13] for an elaborated description of the system.

### Study Setting

In 2019, we deployed the system in three GP offices in Norway, and 20 GPs received a single feedback report on their prescribing of antibiotics for selected RTIs. The GPs were then asked to fill out a web-based questionnaire for assessing their perceptions of the feedback received and how the feedback was presented.

Norwegian GPs are responsible for treating a set of patients and refer only those who need more specialized health services to hospitals [19]. Therefore, the 20 GPs had a total of around 19,345 patients on their lists. The number of registered patients varied during the study period because patients sometimes change their GP.

### Audits and Feedback

The GPs received a single feedback report on their prescribing of antibiotics for selected RTIs for which antibiotics are generally not recommended if the patient is otherwise healthy (eg, during the first presentation of a current episode of an RTI or when the patient has no significant underlying comorbidity) [20]. Based on the *International Classification of Primary Care, Second Edition* [21], the selected RTIs were acute upper respiratory infection (R74), acute sinusitis (R75), acute laryngitis/tracheitis (R77), acute otitis media/myringitis (H71), and unspecified respiratory infections (R83). Acute bronchitis (R78) was also included because it is most often a viral infection, and the use of an antibiotic is rarely recommended.

Feedback was provided for the selected RTI cases between 2015 and 2018; both combined and separate feedback were provided for each of the diagnoses. All statistics were stratified by both year and antimicrobial spectrum (broad- and narrow-spectrum antibiotics). Based on the Anatomical Therapeutic Chemical classification system [22], narrow-spectrum antibiotics include β-lactamase–sensitive penicillins (J01CE), and broad-spectrum antibiotics include tetracyclines (J01A); β-lactam antibacterials and penicillins (J01C, excluding J01CE); other β-lactam antibacterials (J01D); sulfonamides and trimethoprim (J01E); macrolides, lincosamides, and streptogramins (J01F); and quinolone antibacterials (J01M).

The feedback report contains the number of cases that were diagnosed and treated with antibiotics. A set of performance indicators was presented as a time series graph that compares the performance indicators of a GP with the average indicators of all participating GPs. Performance indicators were based on the indicators proposed by the European Surveillance of Antimicrobial Consumption Network [23], as follows:

- The percentage of diagnosed cases treated with an antibacterial drug for systemic use
- The percentage of cases treated with narrow-spectrum antibiotics among all diagnosed cases treated with antibiotics
- The percentage of cases treated with broad-spectrum antibiotics among all diagnosed cases treated with antibiotics

### Questionnaire

A panel of experts with clinical and medical informatics backgrounds developed the questionnaire. As shown in Multimedia Appendix 1, the questionnaire broadly covers aspects that were derived from the following three theoretical constructs of the Theory of Planned Behavior [24]: (1) attitude toward privacy and the prescribing of antibiotics, (2) subjective norms (reflection on the accuracy and appropriateness of using feedback as the basis for comparisons with peers), and (3) self-efficacy regarding changing prescribing behavior.

### Statistical Analysis

Descriptive statistics were calculated to summarize the results of the categorical questions, including age groups and years of experience.

### Ethical Considerations

This study was based on an anonymous questionnaire and therefore did not require ethics review board approval, according to Norwegian regulations. However, all participants gave their informed consent to participate in this study.

## Results

### Participants

**Figure 1:**
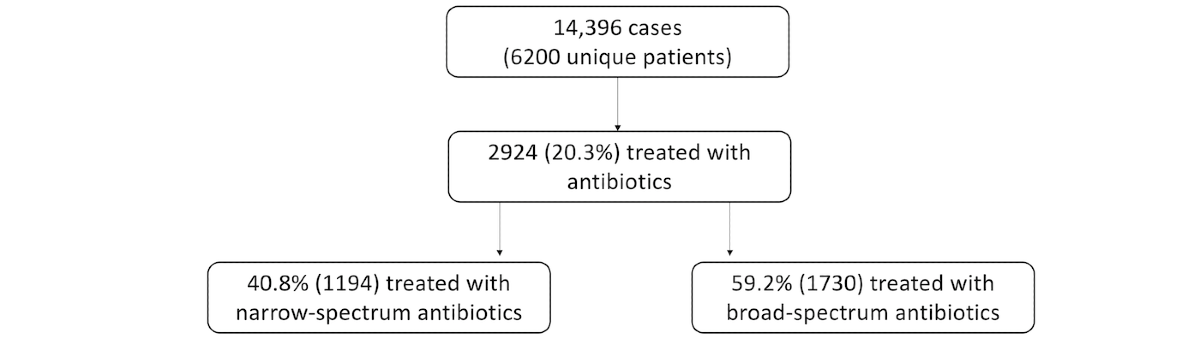
Antibiotic prescribing for all participating general practitioners between 2015 and 2018.

The retrospective data that were generated by using the A&F system show that approximately 20% (2924/14,396, 20.3%) of all cases of the selected RTIs were treated with antibiotics, as illustrated in Figure 1.

A total of 14 GPs responded to the questionnaire, representing a 70% (14/20) response rate. Of these GPs, 36% (5/14) were aged 50 years or older, 57% (8/14) had more than 15 years of experience as a GP, and 79% (11/14) had a specialization in family medicine. The survey results on attitudes toward privacy, audits, and feedback are summarized in Table 1.

**Table 1:** Participant attitudes toward privacy, audits, and feedback (N=14).

	Very useful, n (%)	Useful, n (%)	Not useful, n (%)
Participant attitudes toward patients’ privacy	10 (71)	4 (28)	0 (0)
Participant attitudes toward physicians’ privacy	3 (21)	6 (42)	5 (35)
Participant attitudes toward feedback on own prescribing of antibiotics	9 (64)	4 (28)	1 (7)
Participant attitudes toward comparison with peers	6 (42)	7 (50)	1 (7)

### Privacy Protection

We can observe from Table 1 that the GPs were unanimous about privacy protection for their patients, and 64% (9/14) of GPs considered the protection of their own privacy to be very useful or useful.

### Feedback Report

Most of the GPs (13/14, 92%) reported that feedback on their prescribing of antibiotics and the ability to compare their performance indicators with those of their peers were useful or very useful. The clinicians preferred to receive feedback at regular intervals, such as half-yearly (5/13, 38%) and yearly (7/13, 53%; 1 response was invalid). Most of the clinicians (13/14, 92%) preferred to receive an encrypted feedback report through email rather than having it integrated into the EHR (3/14, 21%), receiving it on a secure website (1/14, 7%), or receiving it on a mobile app (0/14, 0%).

### Effects of Audits and Feedback

Of the 14 GPs, 1 (7%) indicated the intention to change their antibiotic prescribing behavior based on the feedback received, 2 (14%) indicated that they did not intend to change, and the rest (n=11, 78%) were unsure of whether they would change.

### Feedback Stratification

Most of the GPs (11/14, 78%) wanted their feedback to be stratified by the characteristics of their patient populations and diagnoses. Age and gender were the commonly requested patient characteristics for stratification. The clinicians also requested stratification by chronic conditions, such as chronic obstructive pulmonary disease, asthma, cystic fibrosis, other airway comorbidities, acne, perioral dermatitis, and heart failure.

## Discussion

### Privacy Protection

Perhaps the major highlight of this study relates to privacy preservation, since the A&F system resolves most of the privacy concerns raised in similar studies. Our results show that the GPs value the protection of their own privacy as well as that of their patients, and this finding is in line with those of existing studies [25]. Our results appear to support our initial assumption that protecting the privacy of clinicians may increase their willingness to participate in quality improvement activities.

GPs in Norway often work in small practices with few or no peers; therefore, comparisons with peers across multiple institutions could be especially useful, and the privacy safeguards serve as further incentives for participation.

### Feedback Report

Our results show that the GPs were in favor of receiving encrypted feedback reports through their emails. This contrasts with the common belief that integrating a clinical decision support system with EHR systems is an important success factor [26]. It is plausible that the GPs assumed that integration with the EHR would trigger distractive feedback alerts more frequently than those triggered by the desired half-yearly or yearly periodic feedback.

### Effects of Audits and Feedback

The GPs appeared to value both seeing their antibiotic prescribing statistics and being able to compare these with those of their peers. Although this study did not measure whether the clinicians actually changed their prescribing behavior following feedback, it is likely that most of the participants had a close-to-average prescribing practice and would therefore not have an incentive to change their prescribing behavior. In a larger sample of clinicians, there would be a higher number of individuals that deviate from the mean and have an incentive to change their prescribing behavior.

### Feedback Stratification

The GPs requested the stratification of feedback by patient characteristics, such as demographics and chronic diseases, of which both are known to influence the decision to prescribe antibiotics. Stratification provides important information to clinicians on whether their prescribing behavior can be justified based on patient characteristics and enables peer comparisons among clinicians with similar patient populations.

### Limitations

An important limitation is that we could not evaluate GPs’ responses based on their clinical performance, since the questionnaire was anonymous and access to feedback reports was limited to the respective clinician. For example, we were not able to assess how the self-reported intention to change related to an individual’s current clinical performance.

Another limitation is that the feedback reports provided to GPs were not adjusted for comorbidities and age. These two variables are known to influence the decision to prescribe antibiotics, and this may have affected their responses to the survey.

We also noted that the average performance of peers might be skewed if there are outlier clinicians who are far from the mean on 1 side. Therefore, it might be necessary to exclude outliers or provide more statistics, such as SDs and percentiles, to enable detailed comparisons with peers.

The sample size in this study can be considered small but can also be viewed as appropriate for an in-depth case analysis for a pilot implementation of this type.

### Recommendations, Implications for Practice, and Impacts

The findings from this study have important implications for practice, especially implications related to quality improvement programs at health care institutions. We single out 3 elements that we recommend for improving systems that support antibiotic stewardship programs. The first and basic element is protecting the privacy of clinicians as well as that of patients. Second, scalability should be considered so as to enable comparisons among peers across multiple health care providers. Comparisons across health care providers are especially useful for small practices like GP offices, since such comparisons require a wide pool of similar patient groups and can be adjusted for comorbidities and age. Finally, human factors should be considered. For example, the frequency of feedback should be considered, since clinicians may not want distractive feedback alerts.

In terms of impact, our system offers a solution to key challenges that hamper antibiotic stewardship programs. It provides privacy guarantees to patients, clinicians, and health care institutions and provides the scalability required to ensure that long-term audits and feedback can be sustainable parts of stewardship programs.

The impact for Norway might be limited, since Norway is a small country with relatively lower antibiotic prescribing rates compared to those of other European countries [27]. For larger countries with differently organized general practices, a higher effect can be expected, since audits and feedback are the most effective in places where the baseline performance is poorer compared to that of the best general practices [7].

### Conclusion

Our results show generally positive sentiments among GPs regarding the potential impact of our system, but it should be noted that this was an initial study with a relatively small number of participants. Therefore, the absolute number of clinicians with very high prescribing rates was likely to be low. In larger samples, we expect that a higher number of physicians would receive an incentive to change their practice.

## Appendix

Multimedia Appendix 1 Questionnaire.

## Abbreviations

A&F: audit and feedback
EHR: electronic health record
GP: general practitioner
RTI: respiratory tract infection
