# Supplementary material for: A Privacy-Preserving Audit and Feedback System for the Antibiotic Prescribing of General Practitioners: Survey Study
Source: JMIR Form Res. 2022 Jul 13;6(7):e31650. doi: 10.2196/31650 (PMC9330202; doi:10.2196/31650)
Supplement: Multimedia Appendix 1 [file formative_v6i7e31650_app1.doc]

# A privacy-preserving audit and feedback on antibiotic prescribing

You have received a feedback report on your antibiotic prescribing that was generated from the data extracted from the electronic medical record (EMR) in your general practitioner (GP) office. The report compares your antibiotics prescriptions statistics for selected respiratory infections with the average prescriptions of 20 GPs in 3 GP offices including your GP office.

The report was generated by the learning health care system project at the Norwegian Centre for E-Health Research (hereafter NSE), University Hospital of North Norway (UNN). The purpose of the survey is to get your opinions and preferences on the antibiotic prescriptions report you have received through email. Your response to this survey will be collected anonymously, and the responses from all participants will be summarized and used in our publications. We will make sure the identity of individual respondents cannot be inferred on our publications. Please see the document attached with the invitation email for more information about the survey.

The questions are intended to be answered after reading the feedback report. Open ended questions can be answered either in English or Norwegian.

* Required

1. I give my consent for the collection and analyses of my responses to this survey by the NSE. *

|  | Yes |
| --- | --- |
|  | No |
|  |  |

2. How old are you? *

|  | <40 years |
| --- | --- |
|  | 40 – 49 years |
|  | 50 – 59 years |
|  | ≥ 60 |

3. How many years have you worked as a GP? *

|  | ≤ 5 years |
| --- | --- |
|  | 5 - 15 years |
|  | ≥ 15 years |

4. Are you a specialist in general practice? *

|  | Yes |
| --- | --- |
|  | No |
|  |  |

5. I intend to change my antibiotics prescription patterns in the future as a result of the feedback report. *

We would like to know if the feedback report gave you relevant information that will influence your future decisions whether to prescribe antibiotics or not.

|  | Yes |
| --- | --- |
|  | Not sure |
|  | No |

6. Please rate the usefulness of the following attributes of the report. *

|  | Very useful | Useful | Not useful |
| --- | --- | --- | --- |
| My patients' privacy is protected |  |  |  |
| My private information, including  antibiotics prescriptions is not seen  by anyone |  |  |  |
| I get an overview of my own antibiotics prescriptions |  |  |  |
| I am able to compare myself with  other peers |  |  |  |

7. Please comment on the accuracy of the data composing your report. [You may answer either in English or Norwegian]

We would like to know whether your statistics in the report look accurate. Data accuracy is the degree to which data correctly reflects the real event being described. Your indicators were generated based on data extracted from the electronic medical record (EMR) system in your GP office. Data inaccuracy may have occurred during data entry into the EMR or during data processing.

8. Would you like to see your statistics grouped by patients' characteristics? * For example, age ; gender; and disease, such as chronic obstructive pulmonary disease (COPD)

|  | Yes |
| --- | --- |
|  | No |

9. If you answer yes to the previous question, please suggest patients' characteristics of interest for grouping your statistics. [You may answer either in English or Norwegian]

10. Do you have other suggestions on how the report could be improved? [You may answer either in English or Norwegian]

11. How often would you like to receive feedback on your antibiotics prescriptions? *

|  | Every month |
| --- | --- |
|  | Every quarter of a year |
|  | Every six months |
|  | Every year |
|  | Never |
| Other |  |

12. Please indicate the communication channel through which you prefer to receive future reports. *

|  | Email (I receive encrypted reports through email and the keys for opening the reports through SMS) | |
| --- | --- | --- |
|  | Secure web site (I access all my reports on a secure web site) | |
|  | Mobile app (I access all my reports on a secure mobile app) | |
|  | Integrated into the electronic medical record (EMR) system in your office | |
| Other |  |  |
